# Association Between Amygdala Volume and Trajectories of Neuropsychiatric Symptoms in Alzheimer's Disease and Dementia With Lewy Bodies

**DOI:** 10.3389/fneur.2021.679984

**Published:** 2021-07-07

**Authors:** Alberto Jaramillo-Jimenez, Lasse M. Giil, Diego A. Tovar-Rios, Miguel Germán Borda, Daniel Ferreira, Kolbjørn Brønnick, Ketil Oppedal, Dag Aarsland

**Affiliations:** ^1^Centre for Age-Related Medicine (SESAM), Stavanger University Hospital, Stavanger, Norway; ^2^Faculty of Health Sciences, University of Stavanger, Stavanger, Norway; ^3^Grupo de Neurociencias de Antioquia, School of Medicine, Universidad de Antioquia, Medellín, Colombia; ^4^Grupo Neuropsicología y Conducta, School of Medicine, Universidad de Antioquia, Medellín, Colombia; ^5^Semillero de Investigación SINAPSIS, School of Medicine, Universidad de Antioquia, Medellín, Colombia; ^6^Semillero de Investigación NeuroCo, School of Medicine and Engenieering, Universidad de Antioquia, Medellín, Colombia; ^7^Department of Clinical Science, University of Bergen, Bergen, Norway; ^8^Department of Internal Medicine, Haraldsplass Deaconess Hospital, Bergen, Norway; ^9^Universidad Del Valle, Grupo de Investigación en Estadística Aplicada - INFERIR, Faculty of Engineering, Santiago De Cali, Valle Del Cauca, Colombia; ^10^Universidad Del Valle, Prevención y Control de la Enfermedad Crónica - PRECEC, Faculty of Health, Santiago De Cali, Valle Del Cauca, Colombia; ^11^Semillero de Neurociencias y Envejecimiento, Medical School, Ageing Institute, Pontificia Universidad Javeriana, Bogota, Colombia; ^12^Division of Clinical Geriatrics, Center for Alzheimer Research, Department of Neurobiology, Care Sciences and Society, Karolinska Institutet, Stockholm, Sweden; ^13^Department of Radiology, Mayo Clinic, Rochester, MN, United States; ^14^Stavanger Medical Imaging Laboratory, Department of Radiology, Stavanger University Hospital, Stavanger, Norway; ^15^Department of Electrical Engineering and Computer Science, University of Stavanger, Stavanger, Norway; ^16^Department of Old Age Psychiatry, Institute of Psychiatry, Psychology, and Neuroscience, King's College London, London, United Kingdom

**Keywords:** magnetic resonance imaging, amygdala, neuropsychiatric symptoms, Alzheimer's disease, dementia with lewy bodies

## Abstract

**Introduction:** The amygdala is implicated in psychiatric illness. Even as the amygdala undergoes significant atrophy in mild dementia, amygdala volume is underexplored as a risk factor for neuropsychiatric symptoms (NPS).

**Objective:** To analyze the association between baseline amygdala volume and the longitudinal trajectories of NPS and cognitive decline in Alzheimer's disease (AD) and dementia with Lewy bodies (DLB) over 5 years.

**Methods:** Eighty-nine patients with mild dementia were included (AD = 55; DLB = 34). Amygdala volume was segmented from structural magnetic resonance images (sMRI) using a semi-automatic method (Freesurfer 6.0) and normalized by intracranial volumes. The intracranial volume-normalized amygdala was used as a predictor of the Neuropsychiatric Inventory (NPI) total score, ordinal NPI item scores (0 = absence of symptoms, 1–3 = mild symptoms, ≥4 = clinically relevant symptoms), and Mini-Mental State Examination (MMSE) as measured annually over 5 years using gamma, ordinal, and linear mixed-effects models, respectively. The models were adjusted for demographic variables, diagnosis, center of sMRI acquisition, and cognitive performance. Multiple testing-corrected *p*-values (*q*-values) are reported.

**Results:** Larger intracranial volume-normalized amygdala was associated with less agitation/aggression (odds ratio (OR) = 0.62 [0.43, 0.90], *p* = 0.011, *q* = 0.038) and less MMSE decline per year (fixed effect = 0.70, [0.29, 1.03], *p* = 0.001, *q* = 0.010) but more depression (OR = 1.49 [1.09, 2.04], *p* = 0.013, *q* = 0.040).

**Conclusions:** Greater amygdala volume in mild dementia is associated with lower odds of developing agitation/aggression, but higher odds of developing depression symptoms during the 5-year study period.

## Introduction

Among structural imaging markers for neurodegenerative dementias, the hippocampal volume has been extensively investigated in Alzheimer's disease (AD) and dementia with Lewy bodies (DLB) (1, 2). Clinical correlates of hippocampal atrophy include disease progression and decline in various cognitive functions, for instance, episodic memory impairment in AD (3, 4). However, much less is known about medial temporal lobe structures of the limbic system such as the amygdala, localized in the anterior portion of the medial temporal lobe (5, 6).

Multicenter studies using structural magnetic resonance imaging (sMRI) have reported that the extent of the atrophy in the amygdala seems to be comparable to hippocampal atrophy in patients with mild AD (7). Nevertheless, the amygdala volume has received less attention than hippocampal volume, even as amygdala volume also predicts AD progression (8), and is associated with cognitive performance (9). Importantly, the amygdala is an essential structure for emotion regulation and is part of the salience neural network (9). Of note, several systematic reviews and meta-analyses have linked amygdala structure and function with psychiatric disorders such as major depressive disorder, depression with comorbid anxiety, schizophrenia, and bipolar disorder (10–13). However, the role of the amygdala as a potential marker of neuropsychiatric symptoms (NPS) during the course of dementia remains elusive.

A few cross-sectional studies have suggested associations between the amygdala volume and NPS in AD, including aberrant motor behavior (7), irritability, aggression/agitation (14), anxiety (15), and apathy (16). In spite of it, less is known about these relationships in the early stage of dementia. Furthermore, as NPS fluctuate considerably (17, 18), it is essential to evaluate these associations in longitudinal studies. Especially in DLB, this association has not been studied as far as we know. Understanding such association is important to elucidate the role of early patterns of volume reduction that could be relevant markers of the risk of NPS in clinical practice, which could help to implement specific interventions in at-risk patients.

Based on previous reports in AD showing that amygdala lesions are involved in NPS (9, 19), and considering our preliminary findings that NPS are common and fluctuate during the course of AD and DLB (17, 18), here we aimed to analyze the association between amygdala volumes and the longitudinal development of NPS in AD and DLB.

## Materials and Methods

### Study Design and Setting

This is a secondary analysis of the “The Dementia Study of Western Norway” (DemVest) cohort (20). The latter included patients with mild dementia due to different etiologies recruited between 2005 and 2007 across all the dementia clinics in Hordaland and Rogaland counties in Norway. All patients had annual follow-up until death. Exclusion criteria consisted of absence of dementia, moderate or severe dementia, delirium, personal history of bipolar or psychotic disorder, terminal illness, or recently diagnosed major somatic disease. Details of the design, recruitment, and assessments are stated elsewhere (20). The study was approved by the regional ethics committee and the Norwegian authorities. All patients signed informed consent before enrollment in the study.

In the current analysis, we did not include patients with other causes of dementia recruited in the DemVest cohort (*n* = 26) since segmentation of sMRI was only available for AD and DLB patients; more details about imaging availability can be found elsewhere (21).

### Sample

Only subjects with an available baseline high-resolution sMRI were included. Thus, we included a total sample of 89 subjects with mild dementia who had a clinical diagnosis of AD (*n* = 55) or DLB (*n* = 34). Data from the first 5 years (i.e., Baseline + 5 annual follow-ups) of the DemVest study were used in the current study.

### Clinical Assessment

Four clinical specialists independently applied diagnostic criteria. Dementia diagnosis was mainly established using the DSM-IV criteria (22). When non-amnesic features were present, diagnosis of dementia was made through consensus based on the DLB operationalized diagnostic criteria (23). Subjects were further classified as AD (according to the National Institute of Neurological and Communicative Disorders, Stroke-Alzheimer's Disease and Related Disorders Association) (24) or DLB (according to the International consensus criteria) (23). Patients with a Mini-Mental State Examination (MMSE) (25) score ≥20 or a Clinical Dementia Rating scale (CDR) (26) global score equal to 1 were considered to have a mild stage of dementia.

A subset (*AD* = 36; *DLB* = 20) had autopsy confirmation. High congruency between the clinical, imaging, and neuropathological diagnosis was achieved as previously reported (20, 27). Diagnosis was re-assessed regularly during the follow-up by four specialists in geriatric medicine and psychiatry. The final diagnosis (used for analysis in the current study) was based on the last consensus diagnosis, considering clinical and imaging findings but giving priority to neuropathological diagnosis when available.

Annual structured medical evaluations, relevant information regarding past medical history and medical records were used to obtain complete and detailed medical background.

Global cognition was assessed using the MMSE score.

### NPS Assessment

NPS were assessed annually over 5 years with the Neuropsychiatric Inventory (NPI) (28). The NPI was based on the carer report and includes 10 items (in its original version) for various NPS (i.e., Delusions, Hallucinations, Agitation/aggression, Dysphoria/depression, Anxiety, Euphoria/elation, Apathy/indifference, Disinhibition, Irritability/lability, and Aberrant motor behavior). Each symptom is rated in severity (ranging 0–3) and frequency of presentation (ranging 0–4). Thus, multiplying severity by frequency, a resulting item score (or domain score) can be obtained for each symptom (ranging 0–12).

For our analyses, item scores ≥ 4 were considered “clinically relevant symptoms,” item scores between 1 and 3 were considered “mild symptoms,” and item scores = 0 were considered “absence of symptoms” following previously reported methods (17, 18, 29–32). Thus, individual trajectories of NPS were estimated for each of the NPI domains, based on the item scores (and NPI total score) of each patient at each time point of the study (i.e., Baseline + 5 annual follow-ups).

### Amygdala Volumes Segmentation

Baseline sMRI were acquired in three different research centers using 1.5-T scanners at the three involved research centers (Philips Intera in Stavanger and Haugesund, and General Electric Signa Excite in Bergen), with a voxel resolution of 1 × 1 × ≈ 1.5 mm. Detailed sMRI protocols for each center were available elsewhere (21). The sMRI were acquired in coronal planes in the three research centers. Images with artifacts on visual inspection were excluded. The standardized processing pipeline from FreeSurfer 6.0 (http://surfer.nmr.mgh.harvard.edu/) was applied to multicenter sMRI data as follows: movement correction, non-brain tissues removal, automated calculation of Talairach transformation, intensity normalization, subcortical white and gray matter segmentation, cortex boundary tessellation, fully automatic topology correction, and surface deformation to determine CSF/gray matter and gray/white matter boundaries (33–35). For amygdala segmentation and volume calculation, the FreeSurfer 6.0 automated pipeline for subcortical structures segmentation was implemented. A detailed explanation of the FreeSurfer 6.0 subcortical segmentation methods can be found elsewhere (33). Visual inspection of the final Freesurfer amygdala segmentation was conducted by one researcher blinded to diagnosis for accuracy verification. The right and left amygdala volumes of each hemisphere were summed to obtain the total amygdala volume. To control for the effect of head size and gender differences, the intracranial volume-normalized amygdala was estimated as follows: [Total amygdala volume in mm^3^/total intracranial volume in mm^3^] ^*^ 100%, based on previously published evidence and recommendations on studying the amygdala volume in neurodegenerative diseases (36, 37).

### Statistical Analysis

Baseline demographic (i.e., age, gender, and years of education) and sMRI variables were compared between the AD and DLB subgroups using independent samples *t*-test for continuous variables and Chi-square test for categorical variables. Diagnosis-related differences in the intracranial volume-normalized amygdala were adjusted using ANCOVA followed by *post-hoc* pairwise Tukey test given the possible confounder effect of covariates such as gender, age, and center of sMRI acquisition. Non-parametric subgroup differences in clinical variables (i.e., MMSE, and NPI total) were examined using the Mann–Whitney *U*-test.

To assess the trajectories of NPS, each NPI item score (i.e., frequency × severity) was collapsed into an ordinal variable with three levels that indicate the “absence of symptoms” (NPI item score = 0), “mild symptoms” (NPI item score = 1–3), or “clinically relevant symptoms” (NPI item score ≥ 4). Frequency plots for clinically relevant symptoms were created overall and by diagnosis subgroup.

As repeated observations of individuals are not independent (i.e., correlated), and each patient had a subject-specific trajectory of NPS over time (varying not only in the baseline NPS), mixed-effects models were used to estimate the mean of per-person means over 5 years. Mixed-effects models consider the mean population response for ordinal NPI domains over time (fixed effect), as well as the error in that trajectory associated with differences in patient characteristics (random effect). Detailed revision of the application of mixed models in medicine can be found elsewhere (38).

After the exploratory analysis of our dataset, the center of sMRI acquisition was observed as a possible source of variability in demographic and volumetric variables across individuals (supplementary Figures 1, 2 and supplementary tables 1, 2). Therefore, subsequent analyses (i.e., mixed-effects models) included this factor as covariate. Time in the study was also included as a covariate, and the random effects of time were also considered.

For statistical purposes, the intracranial volume-normalized amygdala values were rescaled, multiplying them by 1,000 to avoid the boundary value problem in the subsequent models' estimations.

First, to analyze the associations between amygdala volumes and global NPS in the total sample, we conducted generalized mixed-effects models. In these models, the intracranial volume-normalized amygdala was used as the independent variable and the absolute NPI total score at each time point of the study was used as the dependent variable (added a constant of one to obtain a strictly positive distribution). These generalized mixed-effects models were based on gamma distribution with random intercepts and slopes in an unstructured variance–covariance matrix. Time in study, center of sMRI acquisition, gender, diagnosis, and MMSE total score were included as covariates in a fully adjusted model.

Second, we conducted separate ordinal mixed-effects models using intracranial volume-normalized amygdala as the independent variable and each of the ordinal NPI items (i.e., delusions, hallucinations, agitation/aggression, depression, anxiety, apathy, disinhibition, irritability, and aberrant motor behavior) at each time point of the study as the outcome variable. Euphoria was not included in analyses due to its low frequency and thus lack of power in this study to detect any relevant finding. We again used random intercepts and slopes in an unstructured variance–covariance matrix. All these models were conducted as unadjusted, partly adjusted, and fully adjusted models as follows: The unadjusted models included time and center of sMRI acquisition as covariates. The partly adjusted models also included age, gender, and diagnosis as covariates. The fully adjusted models included the same above covariates as well as longitudinal MMSE scores (i.e., the absolute score of MMSE at each time point of the study). All models included any significant interactions.

Considering potential confounder effects of MMSE on the NPI total and the NPI items estimations, we also examined the associations between intracranial volume-normalized amygdala (independent variable) and the absolute score of MMSE at each study time point (dependent variable) using linear mixed-effects models. Again, we included random intercepts and slopes in an unstructured variance–covariance matrix. This unadjusted model included time and center of sMRI as covariates, and an adjusted model included also age, gender, and diagnosis.

Time interactions with intracranial volume-normalized amygdala were also evaluated in the NPI total score and the MMSE models based on the Akaike Information Criterion (AIC) and the Bayesian Information Criterion (BIC). Significant time ^*^ diagnosis, time ^*^ MMSE, and time ^*^ NPI total score interactions were considered for analysis.

Effect sizes are reported as fixed effects (FE, for NPI total score and MMSE) and odds ratios (OR, for each of the NPI items).

The resulting *p*-values of the predictors in all the above mixed-effects models were corrected for multiple testing using the false discovery rate (FDR) (39). Given the dependence of these *p*-values, we used a tail-area-based FDR (40). The statistical significance level was defined as alpha = 0.05 for all analyses. FDR-adjusted *p*-values (*q*-values) are reported.

Missing data during follow-up, mostly due to death, were assumed to be missing at random, supported by previous publications showing that age, gender, diagnosis, and cognitive decline all predict mortality in this cohort (41).

Additional exploratory analyses for ordinal NPI items, NPI total, and MMSE were conducted stratified by diagnosis. Given the small sample size of each subgroup, these models only included time and center as covariates. Also, considering the exploratory nature of analyses, the resulting *p*-values were not corrected for multiple testing.

Statistical analyses were performed using Stata © version 15.1. RStudio © version 1.1.456 was used for the FDR correction (fdrtool package) and illustrations (ggplot2, seaborn) (40–43).

The current study is reported following the Strengthening the Reporting of Observational Studies in Epidemiology (STROBE) statement (44).

## Results

### Participants

**Figure 1:**
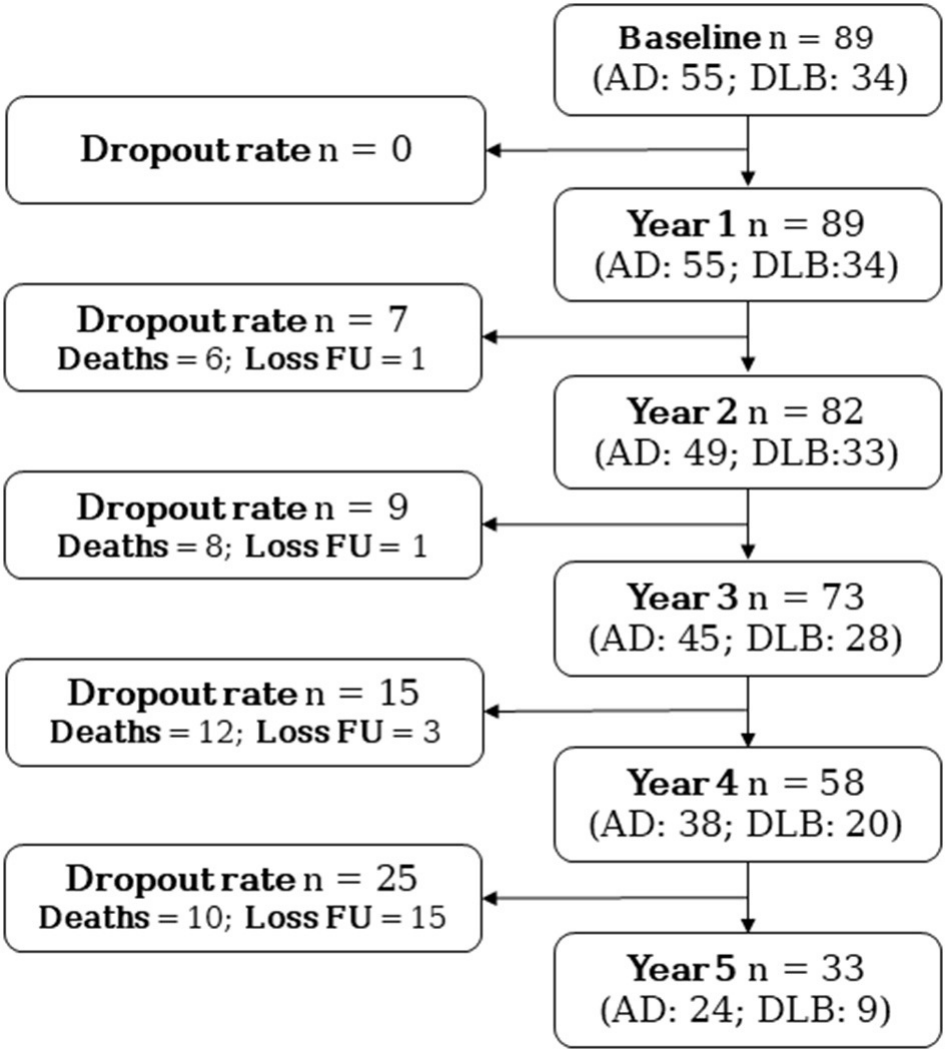
Flowchart of patients at each time point of the study including dropouts. AD, Alzheimer's disease; DLB, Dementia with Lewy bodies; Loss FU, Loss to follow-up.

A total of 89 patients with mild dementia were included (*AD* = 55; *DLB* =34). Demographic and clinical baseline characteristics, as well as the intracranial volume-normalized amygdala, are presented in Table 1. At baseline, age and MMSE were comparable in AD and DLB subgroups. There were significant group differences in gender (*p* < 0.001), where the DLB subgroup had a higher number of men compared to AD. In the DLB subgroup, the baseline NPI total score was higher (*p* = 0.006), but MMSE at baseline was comparable between AD and DLB patients (*p* = 0.966). Figure 1 shows the number of individuals at each time point of the study including dropouts due to death and loss to follow-up. The dropout rate at the latest follow-up examination was 62.9% (*n* = 56), especially due to death, which represents the 64.3% (*n* = 36) among the causes of dropout.

**Table 1:** Baseline characteristics of the AD and DLB subgroups.

	**Total sample (*N* = 89)**	**AD(*N* = 55)**	**DLB (*N* = 34)**	***p*-value**	**Adjusted *p*-value ^b^**
Age	75.4 (7.17)	74.9 (7.28)	76.3 (7.01)	0.383	-
Gender				**<0.001**	**-**
Women (%)	53 (59.55)	41 (74.55)	12 (35.29)	-	-
Men (%)	36 (40.45)	14 (25.45)	22 (64.71)	-	-
Years of education	9.5 (2.79)	9.5 (2.55)	9.4 (3.02)	0.864	-
MMSE ^a^	24 (2.62)	24 (3)	24 (5)	0.966	-
NPI total ^a^	12 (17)	8 (16.5)	16.5 (16)	**0.006**	-
Amygdala (% ICV)	0.154 (0.042)	0.152 (0.045)	0.158 (0.039)	0.487	0.569

*AD, Alzheimer's disease; DLB, Dementia with Lewy bodies; SD, Standard deviation; IQR, Interquartile range; MMSE, Mini-Mental State Examination; NPI, Neuropsychiatric inventory; ICV, Total intracranial volume*.

*Values are presented as mean (SD), and frequency (%) for gender*.

*p < 0.05 are printed in bold*.

a *Values are presented as median (IQR), Mann–Whitney U*.

b *ANCOVA (df = 6), controlling for age, gender, and center of sMRI acquisition with post-hoc Tukey pairwise test by diagnosis*.

### Amygdala Volume at Baseline

**Figure 2:**
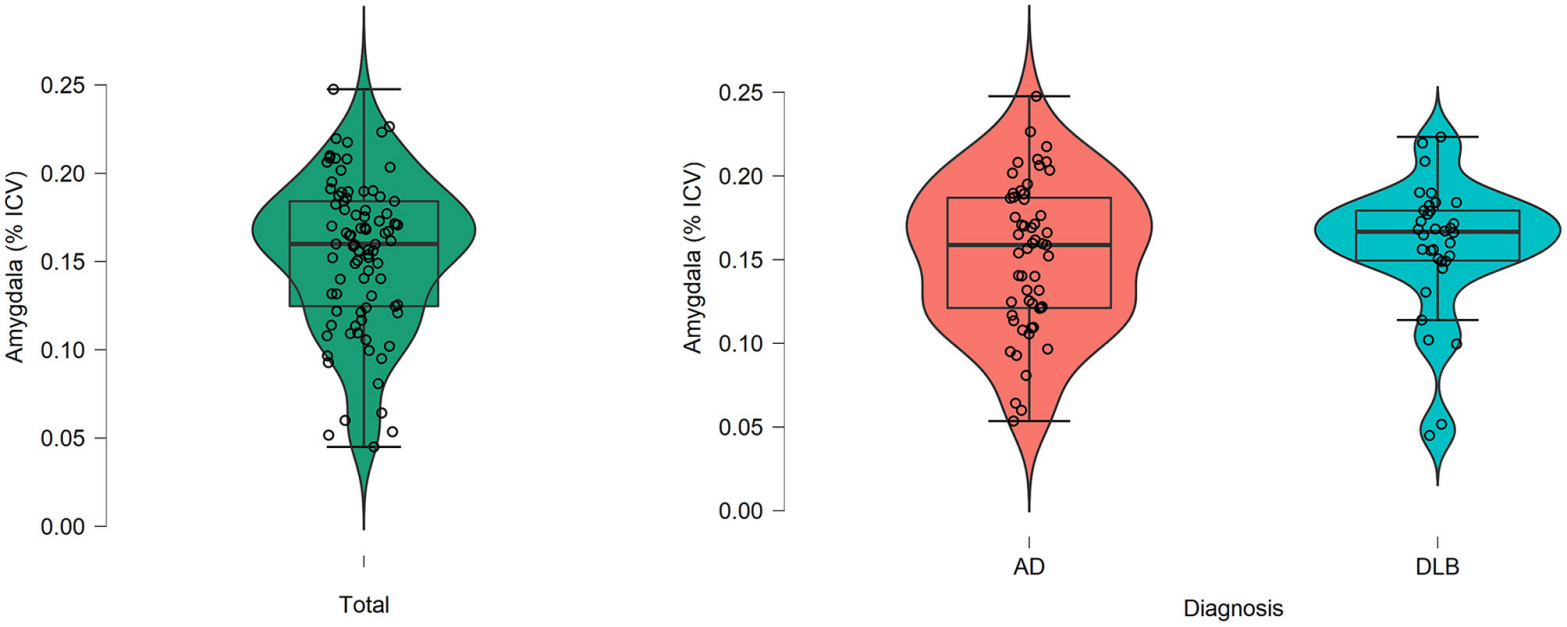
Amygdala volumes after normalization for total intracranial volume in the total sample as well as the AD and DLB groups. Intracranial volume-normalized amygdala (i.e., [Total amygdala volume in mm^3^/total intracranial volume in mm^3^] ^*^ 100%). AD, Alzheimer's disease; DLB, Dementia with Lewy bodies; ICV, Total intracranial volume in mm^3^.

Figure 2 illustrates the values of the intracranial volume-normalized amygdala in the total sample and by diagnosis subgroup. Intracranial volume-normalized amygdala was comparable between the AD and the DLB subgroups (*t* = −0.699, *p* = 0.487). Similar results were obtained when including age, gender, and center of sMRI acquisition as covariates (ANCOVA – *P*_Tukey_ = 0.569) (Table 1).

Significant group differences were observed for the center of sMRI acquisition (*t*-test, *p* < 0.001) but these differences disappeared (ANCOVA, *p* = 0.205) after controlling for age, gender, diagnosis proportion in each center, as well as age ^*^ gender, center ^*^ diagnosis, and center ^*^ age interactions (supplementary Figure 1 and supplementary table 1). Descriptive statistics of the intracranial volume-normalized amygdala by gender are presented in supplementary Figure 2 and supplementary table 2.

### NPS Over Time

**Figure 3:**
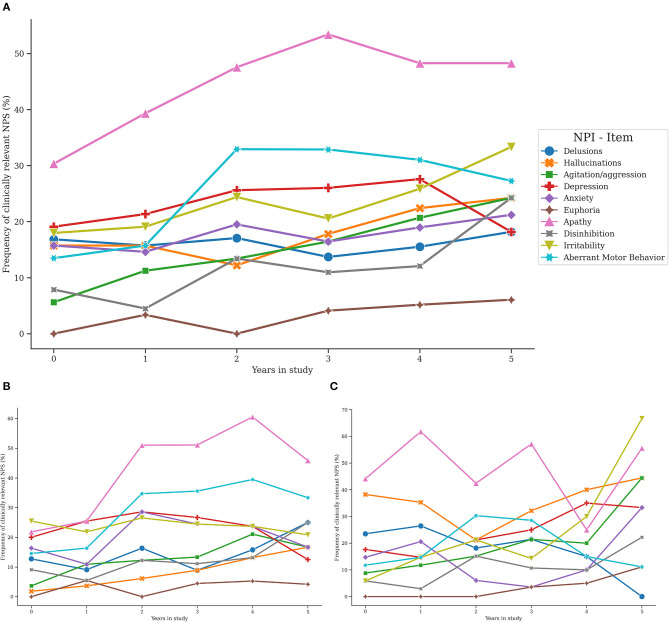
Trajectories of clinically relevant neuropsychiatric symptoms in the total sample, AD and DLB groups. NPS, Neuropsychiatric symptoms; NPI, Neuropsychiatric Inventory; BL, Baseline. Trajectories of clinically relevant NPS in the total sample **(A)**, the AD subgroup **(B)**, and the DLB subgroup **(C)**.

Detailed graphical abstract of the frequency of clinically relevant symptoms is presented in Figure 3, as well as supplementary table 3. Figure 3 depicts the frequency (in percent of symptomatic) of individuals with clinically relevant NPS (i.e., a domain score ≥4) at each time point of the study. Overall, the most frequent symptom in the total sample was apathy.

At baseline, the more frequent clinically relevant symptoms were apathy (30.3%), followed by depression (19.1%) and irritability (18%), whereas euphoria (0%), agitation (5.6%), and disinhibition (7.9%) were the less frequent clinically relevant symptoms. Similarly, at the first annual follow-up, apathy (39.3%), depression (21.4%), and irritability (19.1%) were the more frequent clinically relevant symptoms, while euphoria (3.4%), agitation (11.2%), and disinhibition (4.5%) were the less frequently presented. At the second annual follow-up, apathy was consistently the most presented symptom (47.6%), followed by aberrant motor behavior (32.9%), and depression (25.6%), while clinically relevant euphoria (0%), hallucinations (12.2%), and agitation (13.4%) were the less presented symptoms. In line with this, the higher frequency of presentation at the third annual follow-up was accounted by clinically relevant apathy (53.4%), aberrant motor behavior (32.9%), and depression (26%), but clinically relevant euphoria (4.1%), disinhibition (11%), and delusions (13.7%) were the less frequently presented symptoms. At the fourth annual follow-up, the increasing trend of presenting clinically relevant apathy (48.3%) and aberrant motor behavior (31%) was slightly reduced, but the frequency of clinically relevant depression augmented (27.6%). Besides, clinically relevant euphoria (5.7%), disinhibition (12.1%), and delusions (15.5%) were the less presented symptoms. Finally, at the end of follow-up, clinically relevant apathy (48.3%), irritability (33.3%), and aberrant motor behavior (27.3%) were the more frequent symptoms whereas euphoria (6.1%), delusions (18.2%), and depression (18.2%) were symptoms with the lower frequency of presentation (Figure 3A and supplementary table 3).

In both AD and DLB subgroups, the most frequent symptom during the follow-up was apathy. In DLB patients, the frequency of hallucinations was greater when compared to AD, while aberrant motor behavior was more common in the AD subgroup (Figure 3B, **C** and supplementary table 3).

These results were consistent with previously published analyses of the total DemVest cohort (17, 18).

### Association Between Amygdala Baseline Volume and Trajectories of Global NPS

In an unadjusted model with time and center as predictors, the intracranial volume-normalized amygdala had a significant association with the NPI total score (fixed effect—FE—of −0.14, 95% confidence interval [−0.25, −0.02], *p* = 0.014, *q* = 0.041). Table 2A summarizes the results of the partly adjusted (Model 1) and fully adjusted model (Model 2) for predicting trajectories of the NPI total score in the total sample.

**Table 2A:** Amygdala volume and the Neuropsychiatric Inventory total score over 5 years.

	**Model 1^a^**				**Model 2^a^**			
	**FE**	**95% CI**	***p*-value**	***q*-value**	**FE**	**95% CI**	***p*-value**	***q*-value**
Time	0.14	0.08, 0.19	**<** **0.001**		0.07	0.02, 0.13	**0.010**	
Center^b^								
1	−0.22	−0.56, 0.13	0.223		−0.15	−0.49, 0.19	0.380	
2	−0.47	−0.82, −0.12	**0.008**		−0.44	−0.80, −0.08	**0.017**	
Age	0.03	0.01, 0.04	**0.002**		0.02	0.01, 0.04	**0.006**	
Gender (female)	−0.22	−0.47, 0.03	0.082		−0.21	−0.44, 0.02	0.088	
DLB	0.19	−0.04, 0.42	0.102		0.18	−0.03, 0.39		
MMSE					−0.03	−0.04, −0.02	**<0.001**	
Amygdala (%ICV)	−0.14	−0.25,−0.04	**0.049**	0.091	−0.10	−0.22, 0.01	0.072	0.118

A partly adjusted model showed that the intracranial volume-normalized amygdala had a significant effect over the NPI total score (FE = −0.14 [−0.25, −0.04], *p* = 0.049), but this effect was no longer significant after multiple testing correction (*q* = 0.091). A fully adjusted model did not show significant findings, but a significant effect of MMSE was observed as reported in Table 2A, Model 2.

### Association Between Amygdala Baseline Volume and Trajectories of Various NPS

**Figure 4:**
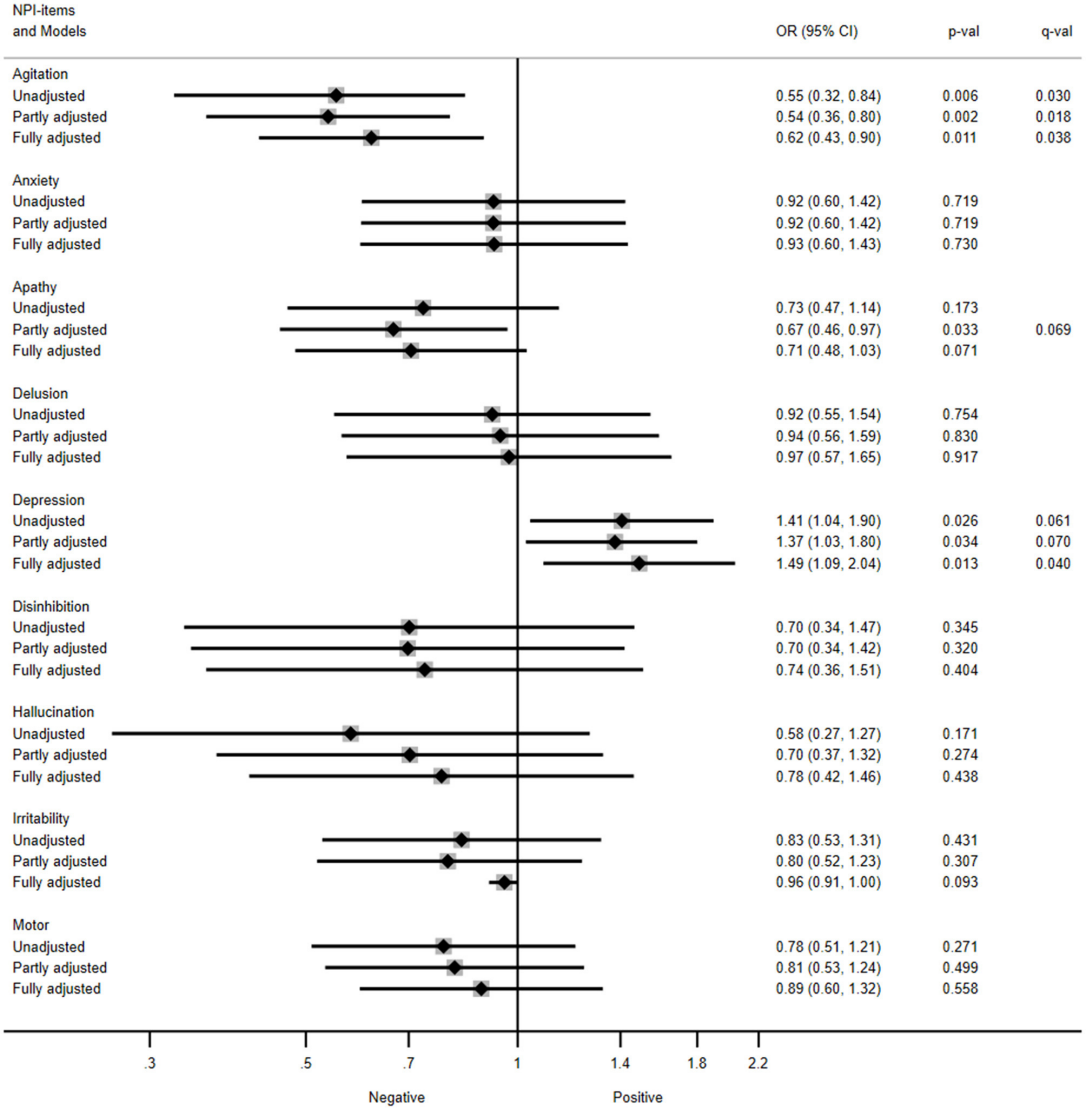
Association between amygdala volume and trajectories of neuropsychiatric symptoms in the total sample. NPI, Neuropsychiatric inventory; OR, Odds ratios; CI, Confidence interval. Unadjusted model included amygdala and time as covariates; partly adjusted model also included age, gender, and diagnosis as covariates; fully adjusted model included MMSE score over 5 years as covariate. The OR (95% CI) refers to the risk of being in a one-level higher group on each of the ordinal NPI items (i.e., presenting mild symptoms, or developing a clinically relevant symptom). Multiple testing-corrected *p*-values (*q*-values) > 0.1 are listed.

Figure 4 summarizes the results of the unadjusted, partly adjusted, and fully adjusted models for predicting trajectories of NPS in the total sample. The odds ratio (OR) refers to the risk of being in a one-level higher group on the ordinal NPI domains (i.e., presenting mild symptoms or developing clinically relevant symptoms).

The unadjusted model showed that patients with higher intracranial volume-normalized amygdala had a reduced odds for presenting mild symptoms of agitation/aggression or developing clinically relevant agitation/aggression over 5 years (OR = 0.55, 95% confidence interval [0.32, 0.84], *p* = 0.006, *q* = 0.030). Similar results were obtained for agitation/aggression in the partly adjusted model (OR = 0.54 [0.36, 0.80], *p* = 0.002, *q* = 0.018), as well as in the fully adjusted model (OR = 0.62 [0.43, 0.90], *p* = 0.011, *q* = 0.038).

Besides, those patients with greater intracranial volume-normalized amygdala had an increased odds for presenting mild symptoms of depression or developing clinically relevant depression as observed in the fully adjusted model (OR = 1.49 [1.09, 2.04], *p* = 0.013, *q* = 0.040). Consistent results were observed in the unadjusted (OR = 1.41 [1.04, 1.90], *p* = 0.026, *q* = 0.061) and partly adjusted models for depression (OR = 1.37 [1.03, 1.80], *p* = 0.034, *q* = 0.070) with a trend to significance in the multiple testing-corrected *q*-values.

In addition, the partly adjusted models showed lower odds for presenting mild symptoms of apathy or developing clinically relevant apathy trajectories in those patients with higher amygdala volumes (OR = 0.67 [0.46, 0.97], *p* = 0.033). However, these results were not significant following correction for multiple testing (*q* = 0.069).

No other NPI domains were significantly predicted by the intracranial volume-normalized amygdala in the main analysis.

### Association Between Amygdala Baseline Volume and Trajectories of Global Cognition

The unadjusted model showed the interaction of intracranial volume-normalized amygdala and time interaction (FE = 0.70 [0.24, 1.16], *p* = 0.003, *q* = 0.020, see Table 2B, Model 3). After adjusting for age, gender, diagnosis, and their significant time interactions, the intracranial volume-normalized amygdala showed a significant FE of 0.66 [0.29, 1.03], *p* = 0.001, *q* = 0.010, Table 2B, Model 4.

**Table 2B:** Amygdala volume and MMSE over 5 years.

	**Model 3^c^**				**Model 4^c^**			
	**FE**	**95% CI**	***p*-value**	***q*-value**	**FE**	**95% CI**	***p*-value**	***q*-value**
Time (T)	−3.01	−3.41, −2.59	**<0.001**		−2.64	−3.08, −2.20	**<0.001**	
Center^b^								
1	0.25	−2.40, 2.90	0.852		1.20	−1.49, 3.91	0.381	
2	−0.32	−2.77, 2.13	0.797		0.16	−2.22, 2.55	0.894	
Age					−0.16	−0.23, −0.09	**<0.001**	
Age × T					0.07	0.01, 0.14	**0.025**	
Gender (female)					0.66	−0.69, −2.01	0.336	
Diagnosis (DLB)					0.74	−0.65, 2.13	0.295	
Diagnosis (DLB) × T					−1.07	−1.99, −1.16	0.022^*^	
Amygdala (% ICV)	0.06	−0.95, 1.07	0.906	NA	0.16	−0.81, 1.12	0.747	NA
Amygdala (% ICV) × T	0.70	0.24, 1.16	**0.003**	**0.020**	0.66	0.29, 1.03	**0.001**	**0.010**

*DLB, Dementia with Lewy bodies; FE, Fixed effect; CI, Confidence interval; MMSE, Mini-mental State Examination; NPI, Neuropsychiatric inventory; ICV, Total intracranial volume; T, Time*.

a *Generalized mixed-effects model with the absolute NPI total score (added a constant of one to provide strictly positive distribution) at each time point of the study as the dependent variable, and intracranial volume-normalized amygdala as independent variable, based on gamma distribution and log-link with random intercepts and slopes in an unstructured variance–covariance matrix with covariates as listed. In a model with time and center as predictors (not in Table), Amygdala (% ICV) had an FE of −0.14 [−0.25, −0.02], p = 0.014, q = 0.041*.

b *Twenty-three participants from reference center (Bergen), 11 in center coded as 1 (Haugesund), 55 in center coded as 2 (Stavanger)*.

c *Linear mixed-effects model with the absolute MMSE score at each time point of the study as the outcome variable, intracranial volume-normalized amygdala as independent variable, random intercepts, and slopes in an unstructured variance–covariance matrix and covariates as listed*.

*Time interactions with intracranial volume-normalized amygdala were also evaluated in the NPI total score and the MMSE models based on the Akaike Information Criterion (AIC) and the Bayesian Information Criterion (BIC). Any significant interaction was included in the above models*.

*p-values and q < 0.05 are printed in bold. Multiple testing corrected p-values (q-values) are listed only for Amygdala (% ICV) (Models 1 and 2), or its interaction with time (Models 3 and 4)*.

**Figure 5:**
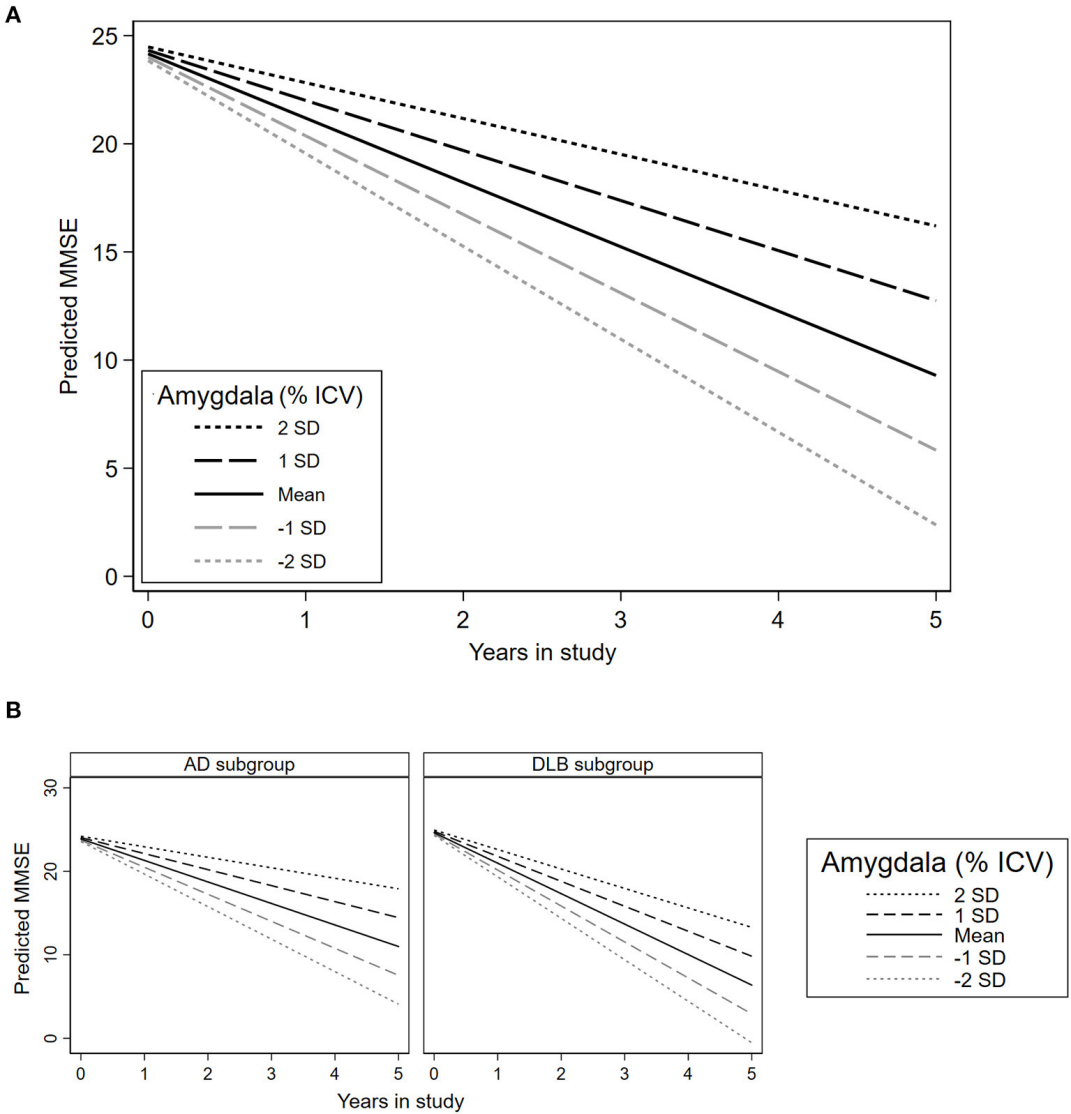
Association between amygdala volume and trajectories of global cognition in the total sample and each diagnosis subgroup. MMSE, Mini-mental state examination; SD: Standard deviation. **(A)** Predictions in the total sample. **(B)** Predictions in the AD and DLB subgroups. This plot is based on the estimations presented in Table 2B, Model 4 (adjusted for time in study, age, gender, diagnosis, and center of sMRI acquisition).

In the total sample, Figure 5A shows the predicted MMSE score over 5 years according to the intracranial volume-normalized amygdala. Patients with higher intracranial volume-normalized amygdala had lesser MMSE decline per year after adjusting for the time in study, center of sMRI acquisition, age, gender, and diagnosis. Consistently, Figure 5B depicts the predicted MMSE score over 5 years according to the intracranial volume-normalized amygdala in both AD and DLB subgroups. In the DLB subgroup, the predicted MMSE score over 5 years according to the intracranial volume-normalized amygdala was lower compared to the AD subgroup.

### Exploratory Analyses by Diagnosis Subgroup

Supplementary Table 4 summarizes the results of the additional exploratory analyses conducted separately in the DLB and AD subgroups. The intracranial volume-normalized amygdala showed a statistically significant effect predicting the MMSE ^*^ time interaction (representing points decline per year per unit of amygdala volume) in AD (*FE* = 0.53 [0.13, 0.93], *p* = 0.010), and stronger in DLB (*FE* = 1.19 [0.31, 2.07], *p* = 0.008). The NPI total was only significantly predicted in the DLB subgroup (*FE* = −0.13 [−0.25, −0.02], *p* = 0.027).

The lower odds of presenting mild symptoms of agitation or developing clinically relevant agitation with higher amygdala volumes were similar in AD (OR = 0.57 [0.36, 0.90], *p* = 0.015) and DLB (OR = 0.47 [0.18, 1.22], *p* = 0.120) but not significant in DLB, likely due to the small sample size. Similar findings were observed in both AD (OR = 1.45 [1.02, 2.06], *p* = 0.041) and DLB groups (OR = 1.38 [0.83, 2.29], *p* = 0.211) for the higher odds of presenting mild symptoms of depression or developing clinically relevant depression.

Interestingly, the DLB subgroup showed a reduced odds of presenting mild symptoms of hallucinations or developing clinically relevant hallucinations in those individuals with a higher intracranial volume-normalized amygdala (OR = 0.49 [0.31, 0.78], *p* = 0.002), which was not observed in AD (OR = 0.94 [0.49, 1.78], *p* = 0.845). Intracranial volume-normalized amygdala was not a significant predictor for other NPS.

## Discussion

This study analyzed the associations between amygdala volume and NPS in AD and DLB, over 5 years of follow-up. We have found that amygdala volumes were comparable between AD and DLB patients at the mild stage of dementia. Greater baseline amygdala volume in patients with mild dementia was associated with lower odds for developing agitation/aggression symptoms. Besides, higher odds for developing symptoms of depression were associated with greater baseline amygdala volume.

The reduction of amygdala volume has been widely studied in AD, but few cross-sectional studies have compared amygdala volume in AD and DLB (45–47), reporting conflicting results (48) that might be attributed to differences in study designs and methods of analysis. In line with our findings, a preliminary study with a matched-sample design combined manual and automatic segmentation and did not find group differences in the amygdala volumes of AD and DLB patients (47). By contrast, two studies found larger amygdala volumes in DLB (45–47), but the lack of adjustment for gender could have influenced their results.

Amygdala atrophy has been demonstrated in moderate and severe AD, but it is also prominent in the early stages of AD (7, 49). Functional and structural brain abnormalities may reflect the neuropathological features of dementia (i.e., Lewy bodies, tau, and amyloid-beta), and a relationship between the burden of Lewy bodies in the amygdala and reduced amygdala volume has been demonstrated in postmortem studies (48). Examining brain volumes in key regions can contribute to the understanding of the structural substrates of different NPS in dementia.

In AD, one longitudinal (2-year study) and several cross-sectional studies suggest close relationships between amygdala volume, cognitive performance, and presence of NPS (7, 9, 19, 49), including agitation/aggression (7, 9, 50). However, none of the available studies have investigated the amygdala volume as an early marker of long-term risk of NPS, and follow-up periods are relatively smaller compared to ours, which can affect the results considering the within-subject fluctuation of NPS over time (17).

We found that baseline amygdala volume had a significant inverse association with the trajectories of agitation/aggression in the total sample, as well as in the AD subgroup. This might reflect damage in circuits underlying behavioral and cognitive avoidance, which could explain clinical features present in agitated/aggressive patients such as overestimation and overreaction to threats, difficulties to modulate strong emotional responses, or increased vigilance and reactivity (9, 50). Aggression circuits involve the amygdala, as well as the insular, anterior cingulate, and orbitofrontal cortexes, which are also described in apathy (9, 19, 50). Thus, an imbalance between the default mode network and the salience network, characterized by increased connectivity in the latter, is hypothesized in those patients with agitation and apathy (9).

An association between baseline amygdala volume and the trajectories of depression was also observed in this study. Of note, this has not been previously reported in dementia patients with longitudinal data. Prior reports in young patients or patients with a first depressive episode linked depression to enlarged amygdala volume (51, 52). Nevertheless, conflicting results also reported an opposite direction for this relationship (53). Large meta-analyses concluded that a direct association between amygdala volume and major depression was particularly observed in depressed patients with comorbid anxiety (10), or those who were under antidepressive treatment (13). In AD, the presence of concomitant Lewy pathology in the amygdala increases the risk for major depression (54). Therefore, longitudinal designs will contribute to filling current knowledge gaps in specific imaging markers of depression in patients with cognitive decline, those with a history of early- and late-onset depression, and healthy older adults.

We adjusted the final models for cognitive decline under the assumption that cognitive deterioration could confound the amygdala volume/NPS association. However, it is equally if not more plausible that cognitive deterioration is an intermediate variable (55), in which case the partially adjusted models would be more appropriate. Therefore, we highlight the findings of the partly adjusted models in agitation/aggression, which are robust across all levels of adjustment and are supported by the available evidence. Also, interesting findings were observed in the partly adjusted models for the trajectories of apathy and depression with a trend to significance after correction for multiple testing.

Previously published cross-sectional evidence has linked apathy and the amygdala (16, 19, 56). In the behavioral variant of frontotemporal dementia, apathy has been considered the result of a disruption of emotional-affective mechanisms functionally linked to the ventromedial prefrontal cortex, the amygdala, and the ventral striatum (57). However, a recent review has pointed out that both emotional-affective and cognitive aspects may lead to impaired motivation and reduced self-generated goal-oriented behaviors that depend on the integrity of cortical and subcortical structures connecting the limbic system and the prefrontal cortex (56). Dopaminergic deficits in the mesolimbic system, especially in the basal amygdala, have also been suggested as a possible underlying mechanism for apathy (58).

Exploratory subgroup analyses showed consistent results in the AD subgroup for agitation and depression. In the DLB group, amygdala volume seems to be associated with the trajectories of hallucinations, but not with other NPS. Remarkably, visual hallucinations have been described as a strong predictor of Lewy pathology; thus, increased burden of Lewy bodies in the amygdala has been reported in DLB patients with visual hallucinations (59).

In conclusion, greater amygdala volume in mild dementia is associated with lower odds of developing agitation/aggression but higher odds of developing depression symptoms during the 5-year study period (particularly in AD). We encourage future works studying NPS in dementia to examine the external validity of our findings.

## Strengths and Limitations

Assessing NPS through a standardized, valid, reliable, and widely available instrument contributes to the reproducibility and external validity of our findings. From the clinical standpoint, defining NPS as “clinically relevant” favors a consistent distinction between overlapping symptoms such as depression and apathy (17); it also facilitates the interpretation of the results. These cutoffs have been widely used in previously published studies (17, 18, 29–32) but have not been validated yet. Conversely, the NPI does not capture the whole spectrum of NPS of dementia and was based on the carer report.

We included patients with a sufficiently long follow-up, considering NPS present from the early to the severe stage of dementia. However, sMRI segmentation from other causes of dementia with remarkable NPS, such as frontotemporal dementia, was not available. In addition, the small sample size and rate of dropouts due to mortality, especially in the DLB group, could lead to underestimations of the trajectories of NPS. Besides, a potential recruitment bias may be present in our data due to the referrals of patients with complicated dementia or NPS from primary care units. However, psychiatric, geriatric, and neurology clinics, as well as GPs, were invited to refer every patient with suspected dementia to minimize this potential source of bias. Also, no control group was available for contrasting our results with normal aging, but clinically relevant NPS were not expected in normal aging (31).

On the other hand, preliminary reports have shown that the amygdala volumes based on the FreeSurfer pipeline have higher validity (i.e., strongly correlations to manual tracing) than other automatic segmentation tools (60, 61). Nevertheless, automatic methods for sMRI segmentation seem to overestimate amygdala volumes, but this does not necessarily imply a lack of validity with adequate visual quality control (61). Also, we are aware of various normalization methods for structural volumes that control for gender differences and head size. However, we conducted amygdala/ICV ratios as it has been demonstrated to control for gender differences in the amygdala volume and facilitate interpretation of the results (36, 37). Further studies with standardized and harmonized normalization protocols can address these limitations. Finally, longitudinal sMRI could be used to estimate atrophy rates and elucidate the potential effects of volume changes over time and the severity of NPS.

## Data Availability Statement

The datasets presented in this article are not readily available because we do not have ethical approval to freely share data to non-related groups. Requests to access the datasets should be directed to Dag Aarsland, daarsland@gmail.com.

## Ethics Statement

The studies involving human participants were reviewed and approved by Stavanger University Hospital. The patients/participants provided their written informed consent to participate in this study.

## Author Contributions

AJ-J: conception of work, visualization, formal analysis, methodology, preparation of the initial draft, writing, reviewing, and approval. LG and DT-R: formal analysis, methodology, writing, reviewing, editing, and approval. MB: conception of work, writing, reviewing, and approval. DF, KB, KO, and DA: supervision, methodology, visualization, writing, reviewing, and approval. All authors contributed to the article and approved the submitted version.

## Conflict of Interest

The authors declare that the research was conducted in the absence of any commercial or financial relationships that could be construed as a potential conflict of interest.

## References

[1] Chow N Aarsland D Honarpisheh H Beyer MK Somme JH Elashoff D. Comparing hippocampal atrophy in Alzheimer's dementia and dementia with lewy bodies. Dement Geriatr Cogn Disord. (2012) 34:44–50. 10.1159/000339727 22922563 PMC3470878

[2] Oppedal K Ferreira D Cavallin L Lemstra AW ten Kate M Padovani A. A signature pattern of cortical atrophy in dementia with lewy bodies: a study on 333 patients from the european dLB consortium. Alzheimers Dement. (2019) 15:400–9. 10.1016/j.jalz.2018.09.011 30439333

[3] Dawe RJ Bennett DA Schneider JA Arfanakis K. Neuropathologic correlates of hippocampal atrophy in the elderly: a clinical, pathologic, postmortem mRI study. PLoS ONE. (2011) 6:e26286. 10.1371/journal.pone.0026286 22043314 PMC3197137

[4] Peng GP Feng Z He FP Chen ZQ Liu XY Liu P. Correlation of hippocampal volume and cognitive performances in patients with either mild cognitive impairment or Alzheimer's disease. CNS Neurosci Ther. (2015) 21:15–22. 10.1111/cns.12317 25146658 PMC6495306

[5] Oler JA. Medial Temporal Lobe, in Encyclopedia of Clinical Neuropsychology, eds Kreutzer J. S. DeLuca J. Caplan B. (New York, NY: Springer New York), 1538–40.

[6] Raslau FD Mark IT Klein AP Ulmer JL Mathews V Mark LP. Memory part 2: the role of the medial temporal lobe. Am J Neuroradiol. (2015) 36:846–9. 10.3174/ajnr.A4169 25414002 PMC7990589

[7] Poulin SP Dautoff R Morris JC Barrett LF Dickerson BC. Amygdala atrophy is prominent in early Alzheimer's disease and relates to symptom severity. Psychiatry Res Neuroimaging. (2011) 194:7–13. 10.1016/j.pscychresns.2011.06.014 21920712 PMC3185127

[8] Rodríguez JJ Noristani HN Verkhratsky A. The serotonergic system in ageing and Alzheimer's disease. Prog Neurobiol. (2012) 99:15–41. 10.1016/j.pneurobio.2012.06.010 22766041

[9] Rosenberg PB Nowrangi MA Lyketsos CG. Neuropsychiatric symptoms in Alzheimer's disease: what might be associated brain circuits? Mol Aspects Med. (2015) 43–44:25–37. 10.1016/j.mam.2015.05.005 26049034 PMC4600424

[10] Espinoza Oyarce DA Shaw ME Alateeq K Cherbuin N. Volumetric brain differences in clinical depression in association with anxiety: a systematic review with meta-analysis. J Psychiatry Neurosci. (2020) 45:406–29. 10.1503/jpn.190156 32726102 PMC7595741

[11] Ho NF Chong PLH Lee DR Chew QH Chen G Sim K. The amygdala in schizophrenia and bipolar disorder: a Synthesis of structural mRI, diffusion tensor imaging, and resting-State functional connectivity findings. Harv Rev Psychiatry. (2019) 27:150–64. 10.1097/HRP.0000000000000207 31082993

[12] Anticevic A Van Snellenberg JX Cohen RE Repovs G Dowd EC Barch DM. Amygdala recruitment in schizophrenia in response to aversive emotional material: a meta-analysis of neuroimaging studies. Schizophr Bull. (2012) 38:608–21. 10.1093/schbul/sbq131 21123853 PMC3329999

[13] Hamilton JP Siemer M Gotlib IH. Amygdala volume in major depressive disorder: a meta-analysis of magnetic resonance imaging studies. Mol Psychiatry. (2008) 13:993–1000. 10.1038/mp.2008.57 18504424 PMC2739676

[14] Wright CI Dickerson BC Feczko E Negeira A Williams D. A functional magnetic resonance imaging study of amygdala responses to human faces in aging and mild Alzheimer's disease. Biol Psychiatry. (2007) 62:1388–95. 10.1016/j.biopsych.2006.11.013 17336945

[15] Davidson RJ. Anxiety and affective style: role of prefrontal cortex and amygdala. Biol Psychiatry. (2002) 51:68–80. 10.1016/S0006-3223(01)01328-2 11801232

[16] Kile SJ Ellis WG Olichney JM Farias S Decarli C. Alzheimer abnormalities of the amygdala with klüver-Bucy syndrome symptoms: an amygdaloid variant of alzheimer disease. Arch Neurol. (2009) 66:125–29. 10.1001/archneurol.2008.517 19139311 PMC2868923

[17] Vik-Mo AO Giil LM Ballard C Aarsland D. Course of neuropsychiatric symptoms in dementia: 5-year longitudinal study. Int J Geriatr Psychiatry. (2018) 33:1361–9. 10.1002/gps.4933 29979473

[18] Vik-Mo AO Giil LM Borda MG Ballard C Aarsland D. The individual course of neuropsychiatric symptoms in people with Alzheimer's and lewy body dementia: 12-year longitudinal cohort study. Br J Psychiatry. (2020) 216:43–8. 10.1192/bjp.2019.195 31506117

[19] Tascone L dos S Bottino CM de C. Neurobiologia dos sintomas neuropsiquiátricos na doença de Alzheimer: uma revisão crítica com foco na neuroimagem. Dement e Neuropsychol. (2013) 7:236–43. 10.1590/S1980-57642013DN70300002 PMC5619193 29213845

[20] Aarsland D Rongve A Piepenstock Nore S Skogseth R Skulstad S Ehrt U. Frequency and case identification of dementia with lewy bodies using the revised consensus criteria. Dement Geriatr Cogn Disord. (2008) 26:445–52. 10.1159/000165917 18974647

[21] Borda MG Jaramillo-Jimenez A Tovar-Rios DA Ferreira D Garcia-Cifuentes E Vik-Mo AO. Hippocampal subfields and decline in activities of daily living in Alzheimer's disease and dementia with lewy bodies. Neurodegener Dis Manag. (2020) 10:357–67. 10.2217/nmt-2020-0039 32967534 PMC7574162

[22] Diagnostic statistical manual of mental disorders: DSM-IV. 4th Edn. Washington, DC : American Psychiatric Association, (1994) Available online at: https://search.library.wisc.edu/catalog/999733358502121

[23] McKeith IG Dickson DW Lowe J Emre M O'Brien JT Feldman H. Diagnosis and management of dementia with lewy bodies: third report of the dLB consortium. Neurology. (2005) 65:1863–72. 10.1212/01.wnl.0000187889.17253.b1 16237129

[24] McKhann G Drachman D Folstein M Katzman R Price D Stadlan EM. Clinical diagnosis of Alzheimer's disease: report of the nINCDS-ADRDA work group? under the auspices of department of health and human services task force on Alzheimer's disease. Neurology. (1984) 34:939–44. 10.1212/WNL.34.7.939 6610841

[25] Folstein MF Folstein SE McHugh PR. “Mini-mental state”. A practical method for grading the cognitive state of patients for the clinician. J Psychiatr Res. (1975) 12:189–98. 10.1016/0022-3956(75)90026-6 1202204

[26] Hughes CP Berg L Danziger WL Coben LA Martin RL. A new clinical scale for the staging of dementia. Br J Psychiatry. (1982) 140:566–572. 10.1192/bjp.140.6.566 7104545

[27] Skogseth R Hortobágyi T Soennesyn H Chwiszczuk L Ffytche D Rongve A. Accuracy of clinical diagnosis of dementia with lewy bodies versus neuropathology. J Alzheimer's Dis. (2017) 59:1139–52. 10.3233/JAD-170274 28731443

[28] Cummings JL Mega M Gray K Rosenberg-Thompson S Carusi DA Gornbein J. The neuropsychiatric inventory: comprehensive assessment of psychopathology in dementia. Neurology. (1994) 44:2308–14. 10.1212/WNL.44.12.2308 7991117

[29] Zhang M Wang H Li T Yu X. Prevalence of neuropsychiatric symptoms across the declining memory continuum: an observational study in a memory clinic setting. Dement Geriatr Cogn Dis Extra. (2012) 2:200–8. 10.1159/000338410 22719746 PMC3379732

[30] Teipel SJ Thyrian JR Hertel J Eichler T Wucherer D Michalowsky B. Neuropsychiatric symptoms in people screened positive for dementia in primary care. Int Psychogeriatrics. (2015) 27:39–48. 10.1017/S1041610214001987 25247664

[31] Lyketsos CG Lopez O Jones B Fitzpatrick AL Breitner J Dekosky S. Prevalence of neuropsychiatric symptoms in dementia and mild cognitive impairment: results from the cardiovascular health study. J Am Med Assoc. (2002) 288:1475–83. 10.1001/jama.288.12.1475 12243634

[32] Aalten P Verhey FRJ Boziki M Bullock R Byrne EJ Camus V. Neuropsychiatric syndromes in dementia: results from the european alzheimer disease consortium: part i. Dement Geriatr Cogn Disord. (2007) 24:457–63. 10.1159/000110738 17986816

[33] Fischl B Salat DH Busa E Albert M Dieterich M Haselgrove C. Whole brain segmentation: automated labeling of neuroanatomical structures in the human brain. Neuron. (2002) 33:341–55. 10.1016/S0896-6273(02)00569-X 11832223

[34] Fischl B van der Kouwe A Destrieux C Halgren E Ségonne F Salat DH. Automatically parcellating the human cerebral cortex. Cereb Cortex. (2004) 14:11–22. 10.1093/cercor/bhg087 14654453

[35] Jovicich J Czanner S Greve D Haley E van der Kouwe A Gollub R. Reliability in multi-site structural mRI studies: effects of gradient non-linearity correction on phantom and human data. Neuroimage. (2006) 30:436–43. 10.1016/j.neuroimage.2005.09.046 16300968

[36] Whitwell JL Crum WR Watt HC Fox NC. Normalization of cerebral volumes by use of intracranial volume: implications for longitudinal quantitative mR imaging. Am J Neuroradiol. (2001) 22:1483–9. 11559495 PMC7974589

[37] Voevodskaya O. The effects of intracranial volume adjustment approaches on multiple regional mRI volumes in healthy aging and Alzheimer's disease. Front Aging Neurosci. (2014) 6:264. 10.3389/fnagi.2014.00264 25339897 PMC4188138

[38] Brown H Prescott R. Applied Mixed Models in Medicine, 3rd Edn. Chichester, England: Wiley Blackwell. (2015).

[39] Storey JD. A direct approach to false discovery rates. J R Stat Soc Ser B Stat Methodol. (2002) 64:479–98. 10.1111/1467-9868.0034630581661

[40] Strimmer K. fdrtool: a versatile r package for estimating local and tail area-based false discovery rates. Bioinformatics. (2008) 24:1461–2. 10.1093/bioinformatics/btn209 18441000

[41] Ibrahim JG Molenberghs G. Missing data methods in longitudinal studies: a review. Test. (2009) 18:1–43. 10.1007/s11749-009-0138-x 21218187 PMC3016756

[42] Wickham H. ggplot2: Elegant Graphics for Data Analysis. (2016) Available online at: https://ggplot2.tidyverse.org

[43] Waskom M. Seaborn: statistical data visualization. J. Open Source Soft. 6(60):3021. 10.21105/joss.03021

[44] Vandenbroucke JP Von Elm E Altman DG Gøtzsche PC Mulrow CD Pocock SJ. Strengthening the reporting of observational studies in epidemiology (STROBE): explanation and elaboration. PLoS Med. (2007) 4:1628–1654. 10.1371/journal.pmed.0040297 17941715 PMC2020496

[45] Burton EJ Karas G Paling SM Barber R Williams ED Ballard CG. Patterns of cerebral atrophy in dementia with lewy bodies using voxel-Based morphometry. Neuroimage. (2002) 17:618–30. 10.1006/nimg.2002.1197 12377138

[46] Barber R Ballard C McKeith IG Gholkar A O'Brien JT. MRI volumetric study of dementia with lewy bodies: a comparison with aD and vascular dementia. Neurology. (2000) 54:1304–9. 10.1212/WNL.54.6.1304 10746602

[47] Hashimoto M Kitagaki H Imamura T Hirono N Shimomura T Kazui H. Medial temporal and whole-brain atrophy in dementia with lewy bodies: a volumetric mRI study. Neurology. (1998) 51:357–62. 10.1212/WNL.51.2.357 9710003

[48] Burton EJ Mukaetova-Ladinska EB Perry RH Jaros E Barber R O'Brien JT. Neuropathological correlates of volumetric mRI in autopsy-confirmed lewy body dementia. Neurobiol Aging. (2012) 33:1228–36. 10.1016/j.neurobiolaging.2010.12.015 21353336

[49] Klein-Koerkamp Y Heckemann R Ramdeen K Moreaud O Keignart S Krainik A. Disease Neuroimaging Initiative for the. Amygdalar Atrophy in Early Alzheimer's Disease. Curr Alzheimer Res. (2014) 11:239–52. 10.2174/1567205011666140131123653 24484275

[50] Trzepacz PT Yu P Bhamidipati PK Willis B Forrester T Tabas L. Frontolimbic atrophy is associated with agitation and aggression in mild cognitive impairment and Alzheimer's disease. Alzheimers Dement. (2013) 9:S95–S104.e1. 10.1016/j.jalz.2012.10.005 23253778 PMC3955297

[51] Frodl T Meisenzahl EM Zetzsche T Born C Jäger M Groll C. Larger amygdala volumes in first depressive episode as compared to recurrent major depression and healthy control subjects. Biol Psychiatry. (2003) 53:338–44. 10.1016/S0006-3223(02)01474-9 12586453

[52] Lange C Irle E. Enlarged amygdala volume and reduced hippocampal volume in young women with major depression. Psychol Med. (2004) 34:1059–64. 10.1017/S0033291703001806 15554576

[53] Omura K Constable RT Canli T. Amygdala gray matter concentration is associated with extraversion and neuroticism. Neuroreport. (2005) 16:1905–8. 10.1097/01.wnr.0000186596.64458.76 16272876

[54] Lopez OL Becker JT Sweet RA Martin-Sanchez FJ Hamilton RL. Lewy bodies in the amygdala increase risk for major depression in subjects with alzheimer disease. Neurology. (2006) 67:660–5. 10.1212/01.wnl.0000230161.28299.3c 16924019

[55] Ananth C V Schisterman EF. Confounding, causality and confusion: the role of intermediate variables in interpreting observational studies in obstetrics hHS public access. Am J Obs Gynecol. (2017) 217:167–75. 10.1016/j.ajog.2017.04.016 PMC5545051 28427805

[56] Pagonabarraga J Kulisevsky J Strafella AP Krack P. Apathy in Parkinson's disease: clinical features, neural substrates, diagnosis, and treatment. Lancet Neurol. (2015) 14:518–31. 10.1016/S1474-4422(15)00019-8 25895932

[57] Quaranta D Marra C Rossi C Gainotti G Masullo C. Different apathy profile in behavioral variant of frontotemporal dementia and Alzheimer's disease: a preliminary investigation. Curr Gerontol Geriatr Res. (2012) 2012:719250. 10.1155/2012/719250 22719755 PMC3376472

[58] Le Heron C Holroyd CB Salamone J Husain M. Brain mechanisms underlying apathy. J Neurol Neurosurg Psychiatry. (2019) 90:302–12. 10.1136/jnnp-2018-318265 30366958 PMC6518466

[59] O'brien J Taylor JP Ballard C Barker RA Bradley C Burns A Collerton D. Visual hallucinations in neurological and ophthalmological disease: pathophysiology and management. J Neurol Neurosurg Psychiatry. (2020) 91:512–19. 10.1136/jnnp-2019-322702 32213570 PMC7231441

[60] Morey RA Petty CM Xu Y Pannu Hayes J Wagner HR Lewis D V. A comparison of automated segmentation and manual tracing for quantifying hippocampal and amygdala volumes. Neuroimage. (2009) 45:855–866. 10.1016/j.neuroimage.2008.12.033 19162198 PMC2714773

[61] Schoemaker D Buss C Head K Sandman CA Davis EP Chakravarty MM. Hippocampus and amygdala volumes from magnetic resonance images in children: assessing accuracy of freeSurfer and fSL against manual segmentation. Neuroimage. (2016) 129:1–14. 10.1016/j.neuroimage.2016.01.038 26824403 PMC7243960

