# Supplementary material for: Association Between Amygdala Volume and Trajectories of Neuropsychiatric Symptoms in Alzheimer's Disease and Dementia With Lewy Bodies
Source: Front Neurol. 2021 Jul 7;12:679984. doi: 10.3389/fneur.2021.679984 (PMC8292611; doi:10.3389/fneur.2021.679984)
Supplement: Supplementary file 1 [file Data_Sheet_1.PDF]

Supplementary Material

Supplementary Figure 1. Intracranial volume normalized amygdala in each research center.

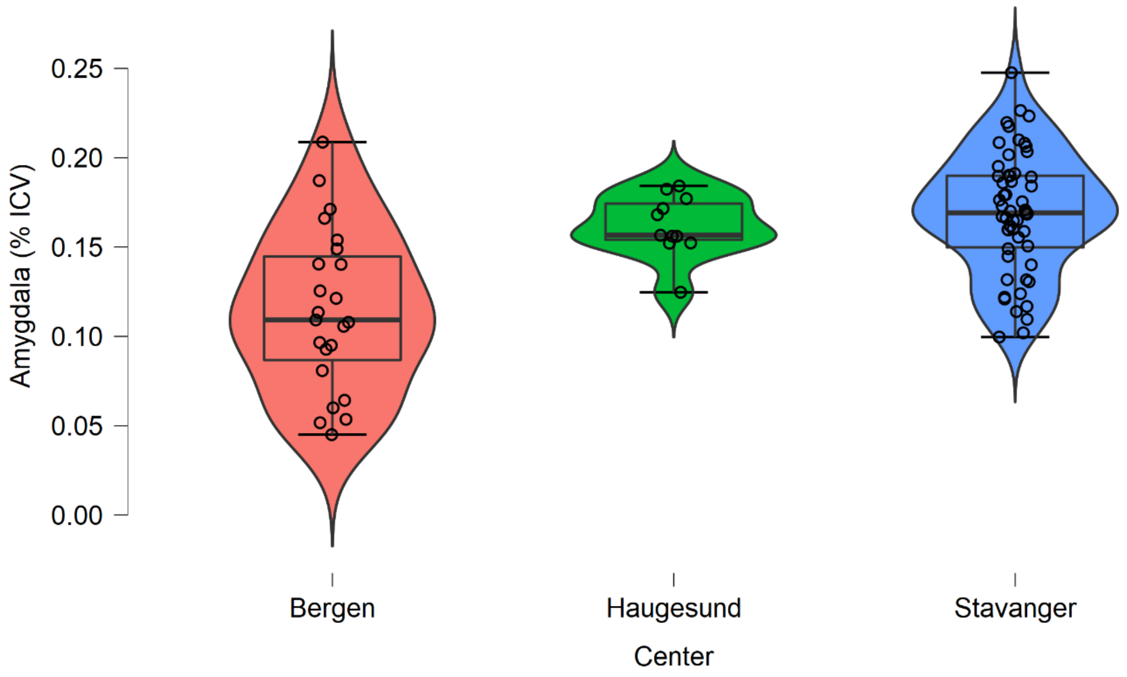

ICV: Total intracranial volume.

Supplementary Figure 1 depicts the intracranial volume-normalized amygdala in each center. In Bergen, the intracranial volume-normalized amygdala (i.e. [Total amygdala volume in mm^3/total intracranial volume in mm^3] * 100%) was lower. Supplementary Table 1 resumes the ANCOVA model conducted in order to control for the effect of possible clinical confounders that might explain the differences across research centers.

Supplementary Figure 2. Age and intracranial volume-normalized amygdala by gender.

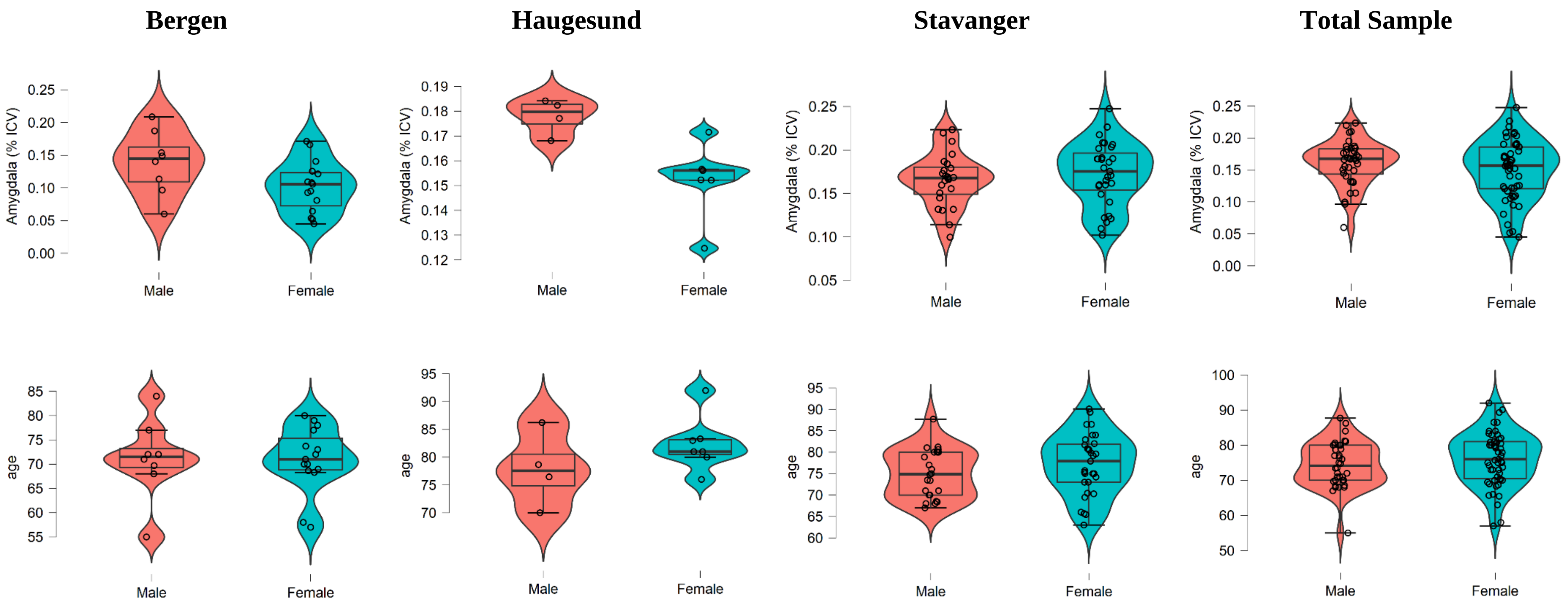

ICV: Total intracranial volume.

Supplementary Table 1. Intracranial-volume normalized amygdala in each research center after controlling for potential confounders

ANCOVA-Total Amygdala/ICV	df	F	p-Value
Center (of sMRI acquisition)	2	1.615	0.205
Diagnosis	1	0.129	0.721
Gender	1	8.345	**0.005**
Age	1	3.741	0.057
Gender * Age	1	7.688	**0.007**
Center * Age	2	0.81	0.449
Center * Diagnosis	2	1.614	0.206

Supplementary Table 1, shows the results of an ANCOVA model using intracranial-normalized amygdala volume as the dependent variable, and center of MRI acquisition as fixed factor. The model was controlled for potential confounders such as age, gender, and diagnosis, as well as gender*age, center*age, and center*diagnosis interactions as covariates. Overall, after controlling for these potential confounders, the results suggested that the intracranial volume-normalized amygdala was comparable between centers (p = 0.205). Thus, differences in the intracranial volume-normalized amygdala might be explained by these clinical factors. In an additional analysis, we have controlled for other potential confounders such as MMSE at baseline and NPI total-score at baseline, but these covariates were not significant.

Supplementary Table 2. Descriptive statistics: Age and the intracranial volume-normalized amygdala by gender.

	Bergen				Haugesund				Stavanger				Total Sample			
	Age (y)		Amygdala (% ICV)		Age (y)		Amygdala (% ICV)		Age (y)		Amygdala (% ICV)		Age (y)		Amygdala (% ICV)	
	Male	Female	Male	Female	Male	Female	Male	Female	Male	Female	Male	Female	Male	Female	Male	Female
Frequency	8	15	8	15	4	7	4	7	24	31	24	31	36	53	36	53
Mean	71.088	70.974	0.139	0.102	77.837	82.330	0.178	0.153	75.050	77.163	0.165	0.172	74.479	76.093	0.161	0.15
Median	71.500	71.000	0.145	0.106	77.560	81.000	0.18	0.156	74.892	77.901	0.168	0.175	74.137	76.000	0.168	0.157
S.D.	8.226	6.689	0.048	0.039	6.693	4.898	0.007	0.014	5.611	7.125	0.031	0.036	6.496	7.588	0.035	0.046
IQR	3.974	6.552	0.053	0.051	5.711	2.653	0.008	0.004	10.000	8.851	0.031	0.043	10.000	10.573	0.039	0.065
Variance	67.661	44.738	0.002	0.002	44.801	23.991	5.196e -5	1.962e -4	31.479	50.764	9.403e -4	0.001	42.199	57.578	0.001	0.002
Minimum	55.000	57.000	0.06	0.045	70.000	76.000	0.168	0.125	67.000	63.000	0.1	0.102	55.000	57.000	0.06	0.045
Maximum	84.000	80.000	0.209	0.171	86.227	92.000	0.184	0.172	87.707	90.085	0.223	0.248	87.707	92.000	0.223	0.248

ICV: Total intracranial volume; S.D: Standard deviation; IQR: Interquartile range.

Supplementary Table 3. Frequency of presentation of clinically relevant NPS over a five-year follow-up

Presenting clinically relevant symptoms - Percent of symptomatic										
**Total sample (n = 89)**										
Years in study	Delusions	Hallucinations	Agitation	Depression	Anxiety	Euphoria	Apathy	Disinhibition	Irritability	AMB
Baseline	16.85	15.73	5.62	19.1	15.73	0	30.34	7.87	17.98	13.48
1	15.73	15.73	11.24	21.35	14.61	3.37	39.33	4.49	19.1	15.73
2	17.07	12.2	13.41	25.61	19.51	0	47.56	13.41	24.39	32.93
3	13.7	17.81	16.44	26.03	16.44	4.11	53.42	10.96	20.55	32.88
4	15.52	22.41	20.69	27.59	18.97	5.17	48.28	12.07	25.86	31.03
5	18.18	24.24	24.24	18.18	21.21	6.06	48.28	24.24	33.33	27.27
**AD subgroup (n = 55)**										
Years in study	Delusions	Hallucinations	Agitation	Depression	Anxiety	Euphoria	Apathy	Disinhibition	Irritability	AMB
Baseline	12.73	1.82	3.64	20	16.36	0	21.82	9.09	25.45	14.55
1	9.09	3.64	10.91	25.45	10.91	5.45	25.45	5.45	21.82	16.36
2	16.33	6.12	12.24	28.57	28.57	0	51.02	12.24	26.53	34.69
3	8.89	8.89	13.33	26.67	24.44	4.44	51.11	11.11	24.44	35.56
4	15.79	13.16	21.05	23.68	23.68	5.26	60.53	13.16	23.68	39.47
5	25	16.67	16.67	12.5	16.67	4.17	45.83	25	20.83	33.33
**DLB subgroup (n = 34)**										
Years in study	Delusions	Hallucinations	Agitation	Depression	Anxiety	Euphoria	Apathy	Disinhibition	Irritability	AMB
Baseline	23.53	38.24	8.82	17.65	14.71	0	44.12	5.88	5.88	11.76
1	26.47	35.29	11.76	14.71	20.59	0	61.76	2.94	14.71	14.71
2	18.18	21.21	15.15	21.21	6.06	0	42.42	15.15	21.21	30.3
3	21.43	32.14	21.43	25	3.57	3.57	57.14	10.71	14.29	28.57
4	15	40	20	35	10	5	25	10	30	15
5	0	44.44	44.44	33.33	33.33	11.11	55.56	22.22	66.67	11.11

AD: Alzheimer's Disease; DLB: Dementia with Lewy bodies; AMB: Aberrant Motor Behavior.

Values represent the frequency (in percent of symptomatic) of individuals with clinically relevant neuropsychiatric symptoms (i.e. NPI domain score ≥4) at each time point of the study.

.

Supplementary Table 4. Amygdala as a predictor of cognition, global NPS, and various NPS in each diagnosis subgroup

	**AD (n = 55)**			**DLB (n = 34)**		
**Outcomes**	**FE**	**95% CI**	**p-val**	**FE**	**95% CI**	**p-value**
**MMSE** [a]	-0.02	-1.18, 1.13	0.967	0.35	-1.51, 2.20	0.716
**MMSE*Time** [a]	0.53	0.13, 0.93	**0.010**	1.19	0.31, 2.07	**0.008**
**NPI-total** [b]	-0.059	-0.22, 0.10	0.468	-0.13	-0.25, -0.02	**0.027**
**NPI items** [c]	**OR**	**95% CI**	**p-val**	**OR**	**95% CI**	**p-value**
**Delusions**	0.88	0.45, 1.73	0.708	1.00	0.69, 1.46	0.983
**Hallucinations**	0.94	0.49, 1.78	0.845	0.49	0.31, 0.78	**0.002**
**Agitation**	0.57	0.36, 0.90	**0.015**	0.47	0.18, 1.22	0.120
**Depression**	1.45	1.02, 2.06	**0.041**	1.38	0.83, 2.29	0.211
**Anxiety**	0.80	0.52, 1.24	0.323	1.07	0.56, 2.02	0.846
**Apathy**	0.80	0.49, 1.30	0.365	0.78	0.46, 1.31	0.345
**Disinhibition**	0.73	0.36, 1.50	0.390	1.07	0.57, 1.98	0.835
**Irritability**	0.90	0.52, 1.53	0.684	0.78	0.44, 1.41	0.419
**Motor**	0.81	0.48, 1.38	0.444	0.77	0.43, 1.38	0.380

AD: Alzheimer's Disease; DLB: Dementia with Lewy bodies; FE: Fixed effects; OR: Odds Ratios; CI: Confidence interval; MMSE: Mini-mental State Examination; NPI: Neuropsychiatric inventory.

[a] Linear mixed-effects model with MMSE as the outcome, random intercepts, and slopes in an unstructured variance-covariance matrix. The intracranial volume-normalized amygdala estimation as the predictor, with time in study and center of sMRI as covariates, is listed for each outcome variable.

[b] Generalized mixed-effects model with NPI-total score as the outcome, based on gamma-distribution and log-link with random intercepts and slopes in an unstructured variance-covariance matrix. The intracranial volume-normalized amygdala estimation as the predictor, with time in study and center of sMRI as covariates, is listed.

[c] Generalized mixed-effects models with each ordinal NPI-item as the outcome with random intercepts and slopes in an unstructured variance-covariance matrix. The estimation of the intracranial volume-normalized amygdala estimation as the predictor of each symptom, with time in study and center of sMRI acquisition as covariates, is listed.

p-Values < 0.05 are printed in bold.
